# The middle house or the middle floor: Bisecting horizontal and vertical mental number lines in neglect

**DOI:** 10.1016/j.neuropsychologia.2007.05.014

**Published:** 2007

**Authors:** Marinella Cappelletti, Elliot D. Freeman, Lisa Cipolotti

**Affiliations:** a Institute of Cognitive Neuroscience, University College London, 17 Queen Square, London WC1N 3AR, UK; b Department of Neuropsychology, National Hospital for Neurology and Neurosurgery, Queen Square, London WC1N 3BG, UK; c Department of Psychology, University of Palermo, via Delle Scienze 15, Palermo, Italy

**Keywords:** Neglect, Number line, Physical line, Bisection, Number cognition, Task-dependence

## Abstract

This study explores the processing of mental number lines and physical lines in five patients with left unilateral neglect. Three tasks were used: mental number bisection (‘report the middle number between two numbers’), physical line bisection (‘mark the middle of a line’), and a landmark task (‘is the mark on the line to the left/right or higher/lower than the middle of the line?’). We manipulated the number line orientation purely by task instruction: neglect patients were told that the number-pairs represented either houses on a street (horizontal condition) or floors in a building (vertical condition). We also manipulated physical line orientation for comparison. All five neglect patients showed a rightward bias for horizontally oriented physical and number lines (e.g. saying ‘five’ is the middle house number between ‘two’ and ‘six’). Only three of these patients also showed an upward bias for vertically oriented number lines. The remaining two patients did not show any bias in processing vertical lines. Our results suggest that: (1) horizontal and vertical neglect can associate or dissociate among different patients; (2) bisecting number lines operates on internal horizontal and vertical representations possibly analogous to horizontal and vertical physical lines; (3) at least partially independent mechanisms may be involved in processing horizontal and vertical number lines.

## 1 Introduction

Unilateral spatial neglect is characterized by the failure to perceive or respond to stimuli located on the side of space opposite to a focal brain lesion (e.g. Driver & Vuilleumier, 2001). One way neglect can manifest is by showing a rightward bias in indicating the midpoint of horizontal lines (e.g. line bisection task, Albert, 1973; landmark task, Bisiach, Ricci, Lualdi, & Colombo, 1998; Milner, Harvey, Roberts, & Forster, 1993). Neglect for vertical lines (altitudinal neglect) has been investigated only in a small number of patients. Altitudinal neglect has been documented as: a upward bias in bisecting vertical lines; a tendency to omit or respond slowly to stimuli presented in the lower part of the space, or slower neglect recovery of the lower quadrant (e.g. Bender & Teuber, 1948; Ergun-Marterer, Ergun, Mentes, & Oder, 2001; Halligan & Marshall, 1991; Ladavas, Carletti, & Gori, 1994; Morris, Mickel, & Brooks, 1986; Pitzalis, Spinelli, & Zoccolotti, 1997; Rapcsak, Cimino, & Heilman, 1988; Shelton, Bowers, & Heilman, 1990). Interestingly, in some of these studies the patients’ performance was more accurate with horizontal than vertical lines (e.g. Ergun-Marterer et al., 2001; Pitzalis et al., 1997; Rapcsak et al., 1988; Shelton et al., 1990), whereas in others vertical lines were better preserved than horizontal lines (Milner & Harvey, 1995; Shelton et al., 1990).

Investigations into neglect have often used visuo-spatial stimuli thus providing insight into the way the brain represents space. However, a few recent neglect studies have also used numbers as stimuli, allowing insight into the way the brain may represent numbers spatially (e.g. Cappelletti & Cipolotti, 2006; Doricchi, Guariglia, Gasparini, & Tomaiuolo, 2005; Priftis, Zorzi, Meneghello, Marenzi, & Umilta’, 2006; Rusconi, Priftis, Rusconi, & Umilta’, 2005; Vuilleumier & Rafal, 1999; Vuilleumier, Ortigue, & Brugger, 2004; Zorzi, Priftis, & Umilta’, 2002; see also Longo & Lourenco, 2007 for a study on healthy participants). It has been proposed that numbers may be represented spatially along a mental line, with smaller numbers located to the left and larger numbers to the right of the line. This proposal has been based on behavioural, neuroimaging and lesion studies. Behavioural studies documented the SNARC effect: healthy subjects asked to classify numbers as odd or even, i.e. ‘parity judgment task’, are faster to judge/classify smaller numbers when responses are made with their hand positioned on the left side of space, but faster to judge larger numbers when responses are made with their hand on the right side of space (Dehaene, Bossini, & Giraux, 1993). This behavioural evidence has recently been corroborated by imaging studies suggesting the involvement of the same brain areas in numerical and spatial transformation tasks (Milner & Goodale, 1995; Simon, Mangin, Cohen, Le Bihan, & Dehaene, 2002) and by a TMS study reporting that the same brain areas are critical for both visuo-spatial search and a number comparison task (Göbel, Walsh, & Rushworth, 2001). In all the above-cited studies the number line has been interpreted as horizontally oriented with left-to-right direction.

Only a few recent studies have investigated performance of patients with neglect when processing numbers, some of these studies in conjunction with physical line bisection. Zorzi et al. (2002) tested neglect patients with a line bisection task requiring them to state the middle number between pairs of numbers. Patients typically stated that ‘4’ was the middle between ‘1’ and ‘5’ (Zorzi et al., 2002). To explain such an effect of biased number bisection, Zorzi et al. (2002) argued in favour of an isomorphism hypothesis between the representation of space and numbers. According to this hypothesis, mental number bisection operates on an internal representation analogous to a horizontal line, with small numbers positioned on the left and large numbers on the right. Therefore, if a patient bisects a physical line towards the right, bisection of the mental number line should also be biased towards the right. A similar interpretation has also been put forward by a subsequent study investigating the explicit and implicit representational space in neglect (Priftis et al., 2006; for a review see also Hubbard, Pinel, Piazza, & Deahene, 2005). However, in Zorzi et al.'s study (2002) no data are reported on the patients’ performance in physical line bisection. This makes it difficult to fully evaluate their proposed isomorphism hypothesis.

In contrast, two recent studies investigated neglect patients’ performance on bisecting both physical and mental number lines. A double dissociation was initially reported by Rossetti and colleagues in two neglect patients (Rossetti et al., 2004). Subsequently, Doricchi et al. (2005) described a rightward bias for physical line bisection but no corresponding bias in mental number line bisection in three patients with both neglect and hemianopia. Conversely, a rightwards bias in mental number line bisection but no shift of physical line bisection was present in three out of eight patients with neglect and no hemianopia. Doricchi et al. (2005) proposed that processing mental numbers along a line required representational mechanisms that are distinct from processing physical line midpoints. They attributed biased performance in bisecting the mental number line to impairment in the spatial working memory mechanisms allowing the navigation along this line. These mechanisms are thought to be underpinned by prefrontal areas (Doricchi et al., 2005). This account assumes that biased performance in mental number line bisection is a type of representational neglect. This proposal is broadly consistent with past accounts suggesting that representational neglect is due to damage to the visuo-spatial component of working memory (Baddeley & Lieberman, 1980; Beschin, Cubelli, Della Sala, & Spinazzola, 1997). It remains unclear, however, why deficits in spatial working memory should manifest as such a specific spatial bias towards the right-hand side in imagery and mental number bisection. In addition, five out of eight patients in Doricchi et al.'s study (2005) showed an associated right bias both in physical and mental number lines. These data are in line with the isomorphism hypothesis, as at least for some patients physical and mental number line bisection were associated in performance, implying that the two hypothesis (isomorphism and spatial working memory) need not be exclusive.

The aim of this study was to further investigate the mechanisms operating in both physical and mental number bisection for horizontal and vertical lines in neglect patients. On the basis of the isomorphism hypothesis we reasoned that wherever joint bias is found both for physical and number line bisection, the pattern of bias should depend on horizontal versus vertical line direction in the same way for both modalities. In contrast, on the basis of the spatial working memory hypothesis it should be possible to document dissociations in patients’ performance when bisecting physical and number lines as a function of their orientation. This is because physical versus mental number line bisection may depend on different processes (visual versus representational) and therefore need not respect the same constraints relative to line orientation.

## 2 Case descriptions

Five patients with unilateral neglect were assessed in the Neuropsychology Department of the National Hospital for Neurology and Neurosurgery in London, UK. All patients gave written informed consent, and the study was approved by the Ethics Committee of the Institute of Neurology in London.

### 2.1 Case 1

**Fig. 1:**
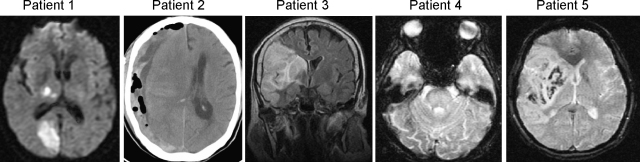
The patients’ brain scan.

Patient 1 was a 64-year-old English-speaking caucasian female who sustained a right posterior cerebral artery territory infarct in January 2006. An MRI-scan showed an area of restricted diffusion affecting the right temporal and occipital lobes and the right thalamus (see Fig. 1A). According to the medical records, there was hemianopia but no sign of optic ataxia or any other visual field deficit.

### 2.2 Case 2

Patient 2 was a 55-year-old English-speaking caucasian male who sustained a subdural hemorrhage affecting the right fronto-parietal regions in October 2005 (see Fig. 1B). The CT scan also showed a marked cerebellar volume loss. According to the medical records, there was no hemianopia and no sign of optic ataxia or any other visual field deficit.

### 2.3 Case 3

Patient 3 was a 69-year-old English-speaking female who sustained a large, acute middle cerebral artery territory infarct. An MRI-scan showed a right hemisphere lesion involving the lentiform nucleus, the right fronto-parietal and temporal cortex consistent with MCA territory infarct (see Fig. 1C). According to the medical records, there was no hemianopia, and no sign of optic ataxia or any other visual field deficit.

### 2.4 Case 4

Patient 4 was a 78-year-old English-speaking caucasian male, who sustained a right middle cerebral artery territory infarct in March 2006. A MRI-scan showed right parieto-occipital lesion in addition to pronounced generalized supra and infratentorial volume loss (see Fig. 1D). According to the medical records, there were no sign of hemianopia, optic ataxia visual or other field deficit.

### 2.5 Case 5

Patient 5 was a 60-year-old English-speaking oriental female who sustained a middle cerebral artery territory infarct involving the basal ganglia, the right posterior frontal and parietal lobe (see Fig. 1E). According to the medical records, there was no hemianopia, and no sign of optic ataxia or any other visual field deficit.

### 2.6 Neuropsychological test findings

All patients were administered neuropsychological tests evaluating general intellectual functioning, memory, picture naming, and executive functions. Visuo-perceptual and visuo-spatial functions and tests for neglect were also performed. The results are reported in Table 1.

Patients showed marked impairment in non-verbal abstract reasoning tasks, the only exception being patient 2 who was able to obtain a reasonable score in the Progressive Matrices. The patients’ performance on the verbal scale of the WAIS-R was relatively preserved in two patients (patients 2 and 4) and impaired in the remaining three (patients 1, 3 and 5). Similarly, they were also all impaired in visual memory functions. In contrast, verbal long-term and short-term memory were well preserved across the patients, the only exception being patient 2 who showed an impairment in verbal memory. Nominal functions were relatively preserved. All patients performed very poorly on the phonemic fluency test (i.e. letter ‘S’), known to be sensitive to frontal lobe disfunction. Visuo-perceptual and visuo-spatial functions were gravely impaired in all patients with only one exception (patient 1).

Dense left unilateral neglect was documented in all patients. In the ‘Star cancellation Test’ patients omitted to cross the majority of the stars on the left-hand side of the paper (Wilson, Cockburn, & Halligan, 1987). In patient 1, left neglect was so profound that she was able to cross only the stars that were on the extreme right-hand side of the paper. This pattern of performance has been previously reported in neglect patients (see Husain & Rorden, 2003). In the ‘Object drawing’ task, patients were able to copy correctly the right side of the pictures (i.e. a star, a cube, a daisy, Wilson et al., 1987). However, they all neglected significant details of the left side. In the ‘line bisection task’ all patients showed evidence of shifting towards the right (Diller, Ben-Yishay, & Gerstman, 1974).

Overall, the cognitive profile of our neglect patients tended to be rather similar, the only exception being the preservation of performance on a non-verbal test of abstract reasoning and on a visual perception task in one patient (patients 2 and 1, respectively), and impairment on verbal memory and on the verbal part of the WAIS-R in one and two patients respectively (patient 4; patients 3 and 5, respectively). All patients presented equally dense neglect, with patient 1 being somewhat more impaired in one particular task (i.e. ‘Star cancellation’).

## 3 Experimental investigation

There were three experimental tasks. Task 1 was a ‘Mental Number Bisection’ where participants were presented with two spoken numbers. There were a horizontal and a vertical condition. In the horizontal condition subjects were asked to think of the numbers as indicating houses along a street; in the vertical condition, they were asked to think about the numbers as floors on a building. Participants were asked to say which number was in the middle of the two orally presented numbers. Task 2 was a ‘physical line bisection’ where participants were asked to put a mark as accurately as possible in the centre of horizontal and vertical lines. Task 3 was an adaptation of the original ‘landmark task’ (Bisiach et al., 1998; Milner et al., 1993; Westheimer, Crist, Gorski, & Gilbert, 2001). Participants had to judge the position of a mark on a line: right or left of the midpoint of horizontal lines; higher or lower of the midpoint of vertical lines. In all the three tasks, participants were seated at a table next to the experimenter, who ensured that their body position remained constant throughout the testing; head and eye movements were unrestricted and no time limit was imposed. Tasks were administered to the participants in different orders to avoid carry-over effects. As the number of patients participating in the study could not be anticipated, task order for patients could not be fully randomised. Therefore, the order was such that for any new patient, the first task was not the same as for the previous patient. For instance, if the first task for the first patient was mental number bisection, the first task for the second patient was physical line bisection. Prior to the beginning of each experiment, ten initial trials were given to the participants for training purposes. These trials were based on a small subset of experimental stimuli and were not included in analysis.

### 3.1 Control subjects

Twelve right-handed volunteers with no history of neurological or psychiatric illness (six males) and matched as closely as possible for age and education to the patients (mean age 59 years, S.D. = 3.2; mean education 14.3 years, S.D. = 2.4) performed Tasks 1 and 2. Six of these control subjects also performed Task 3.

### 3.2 Stimuli and procedure

#### 3.2.1 Task 1: mental number bisection

Task 1 was controlled using the Cogent Graphics toolbox (http://www.vislab.ucl.ac.uk/Cogent/) and Matlab7 software on a S2VP Sony laptop computer. Stimuli consisted of pairs of numbers from 1 to 31 in ascending (e.g. ‘1–5’) or descending order (e.g. ‘5–1’) in four different numerical ranges: 3 (e.g. ‘1–3’), 5 (e.g. ‘1–5’), 7 (e.g. ‘1–7’) and 9 (e.g. ‘1–9’), following Zorzi et al.'s (2002) study. There were 12 ascending and 12 descending pairs of stimuli for each numerical range presented with equal frequency in pseudo-random order. There were 4 blocks with 36 trials each (total = 144). In 2 blocks (72 trials), participants were instructed to imagine the numbers as indicating items oriented horizontally such as houses along a street. For the remaining two blocks, participants were instructed to imagine the numbers as indicating items oriented vertically such as floors in a building. Blocks were presented with ABBA design. Each trial started with a sound presented for 100 ms, and followed by pairs of numbers orally presented through the computer speakers. Participants were asked to say the middle number in each pair; responses were recorded and scored by the experimenter.

#### 3.2.2 Task 2: physical line bisection

Stimuli were 72 horizontally and 72 vertically oriented black lines randomly presented in equal proportion on the four quadrants of an A4 page. Six different lengths were used for each type of line: 2, 3.5, 5, 8, 10, and 15 cm (three trials for each length for each type of line). Each A4 paper was positioned in front of the participants, directly opposite the body midline. The viewing distance was about 50 cm. Participants were asked to mark the middle of each line.

#### 3.2.3 Task 3: landmark

Stimulus presentation and data collection in Task 3 used the same laptop and software as Task 1. The dimensions of the display, as rendered on the built-in liquid-crystal screen, were 23.5 cm horizontal by 18 cm vertical. The display had a resolution of 640 × 480 pixels and was refreshed at a frequency of 60 Hz. Stimuli were white, with luminance of 205 candelas per square metre (cdm^−2^), presented on a mid-gray background of luminance 44 cdm^−2^. Stimuli consisted of long horizontal or vertical white lines. Each long line was bisected at varying positions along its length by short white ‘landmark’ lines, oriented at 90° relative to the long line. From a viewing distance of 50 cm, the long lines subtended a visual angle of 3.2° long and 0.9° wide (56 and 1.5 mm, respectively); the landmark lines were 9.9° long by 0.9° wide (17.5 and 1.5 mm, respectively). These stimuli were presented unpredictably in one of four quadrants of the screen (upper left, upper right, lower left and lower right), in a counterbalanced order with equal frequency. These four possible stimulus positions were fixed at 1.15° eccentricity from central fixation (as if forming the corners of an invisible virtual square). The veridical midpoint of the long line was always centered on one of these four positions.

There were four blocks for horizontal and vertically orientated lines, respectively, presented in alternating order. Each block was composed of a sequence of trials, varying in number depending on the subject's performance (see below). Each trial commenced with a small fixation point in the centre of the screen, which disappeared when the subject pressed the spacebar. Following an inter-stimulus interval of 200 ms, the line stimulus was displayed for 200 ms. Following offset of the stimulus, the screen remained blank until the subject responded. For the horizontal stimuli, subjects indicated whether the landmark appeared left or right of the perceived centre of the line using the left- or right-arrow keys on the laptop keyboard. For the vertical stimuli, subjects indicated whether the landmark appeared higher or lower of the perceived centre of the line using the up- or down-arrow keys. The position of the landmark was initially chosen at random. However it varied on each trial depending on the subject's previous response, according to an adaptive algorithm (Modified Binary Search, or MoBS, Tyrrell and Owens, 1988). This algorithm identifies the subjects’ point of subjective equality (PSE). This is the landmark position at which subjects are equally likely to respond ‘left’ and ‘right’ (for horizontal lines), or ‘up’ and ‘down’ (for vertical lines). See Appendix A for a full explanation of the algorithm. Four interleaved algorithms were used to find the PSE for each of the four quadrant positions independently.

## 4 Analysis of data and statistical tests

For each line in the mental number bisection task (Task 1), the position of the participants’ number bisectors was measured as deviation in integer units from the veridical mid-number. Positive units indicated deviations towards the right or the upper end of the mid-number for horizontal and vertical number lines, respectively. In contrast, negative units indicated deviations towards the left or the lower end of the mid-number for horizontal and vertical number lines, respectively. In Task 2, for each physical line the same criteria used in Task 1 were adopted. The position of the participants’ marked bisectors in the physical line bisection task was measured in centimetres from the veridical midpoint. For example, +2 cm represented an error to the right of the midpoint for horizontal lines or higher than the midpoint for vertical lines. Therefore, neglecting the left-hand side of physical lines horizontally oriented resulted in positive values of deviation. Similarly, neglecting the lower part of physical lines vertically oriented also resulted in positive deviations.

The following effects were measured.

(i) The significance of the bias: non-parametric and parametric tests were used in patients and control subjects respectively to determine whether there were consistent deviations in their bisection performance.
(ii) The increase of the bias as a function of line length: a linear regression analysis was used to examine the relationship between the position of participants’ bisectors (as deviation in integer units or cm from the veridical mid-number or midpoint) and the length of the physical or number line. For each participant, bisectors were averaged across each numerical interval or each line length rather than across the whole interval or length. The slope of the regression lines was also estimated to assess the amount of the bisector deviation increment with every unit increase in terms of number interval or line length.
(iii) Any difference in performance between patients and control subjects on horizontal and vertical dimensions in each task.
(iv) Any difference in bisecting horizontal and vertical lines within each task. Differences between horizontal and vertical dimensions in control subjects may suggest that processing the two dimensions differ in terms of difficulty, familiarity or markedness (e.g. Trask, 1999). To test for these effects, three indices were used in control subjects to compare horizontal and vertical lines in the two tasks: (1) a *t*-test comparing the bias in the two dimensions; (2) a measure of the correlation between them; (3) an analysis of response times in Tasks 1 and 3 [ANOVA with line orientation and length as factors]. Non-parametric tests were used in neglects patients to test for differences between horizontal and vertical lines.
(v) Any difference between the two bisection tasks in both patients and control subjects. For this purpose, as different measures were used for number and physical lines, i.e. units and cm, respectively, we first transformed these values into a common measure, namely we normalised them. This normalisation was obtained by dividing the value of each produced bias by the value of the maximum possible bias for each line. For instance, given a physical line of 2 cm length, the maximum possible value of the bias is 1 cm (positive or negative). Therefore, a bias of 0.4 cm corresponds to a normalised value of 0.4 in a 2-cm line (i.e. 0.4–1) and of 0.16 in a 5-cm line (i.e. 0.4–2.5). Once the normalised values were been obtained for each line length and each numerical range in the two tasks, they were compared using non-parametric tests and *t*-tests.

In Task 3, PSE values were normalised to the range of 0–1, corresponding respectively to left and right line ends (for horizontal lines) or down and up line ends (for vertical lines), with the veridical midpoint being 0.5. The extent to which individual patients’ PSE's differed reliably from the controls was assessed relative to the controls’ group mean and standard error. Statistical reliability of differences between conditions was also assessed for each patient individually by constructing 95% confidence limits for each PSE estimate (derived using a bootstrapping procedure, see Appendix B).

## 5 Results

All five patients were tested on bisection of mental number line with both horizontal and vertical lines (Task 1); three out of these five patients (patients 1, 4, and 5) were also tested on bisection of horizontal and vertical physical lines (Task 2), whereas the remaining two (patients 2 and 3) were only tested on bisection of horizontal physical lines. Finally, two out of the five patients (patients 1 and 4) were tested with the landmark test (Task 3). Not all tests could be administered as some patients were discharged before completing all tasks.

### 5.1 Task 1: mental number line bisection

#### 5.1.1 Horizontal lines

**Fig. 2:**
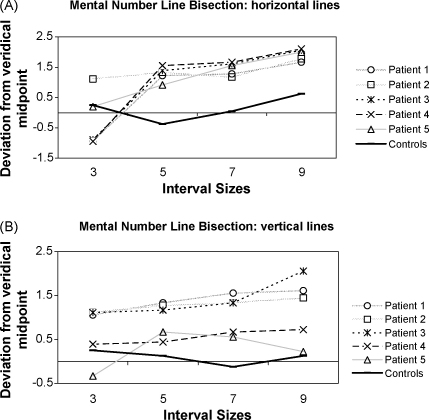
Mental number bisection task (Task 1). Patients’ and control subjects’ deviations from the veridical midpoint in horizontal (A) and vertical (B) number lines in units.

All patients made errors in bisecting mental number lines horizontally oriented (patient 1: 35%; patient 2: 36%; patient 3: 33%; patient 4: 32%; patient 5: 32%). Non-parametric tests indicated that each patient was significantly biased overall towards the right of number lines [patient 1: *Z* = −2.64, *p* < 0.008; patient 2: *Z* = −8.37, *p* < 0.001; patient 3: *Z* = −3.43, *p* < 0.001; patient 4: *Z* = −3.65, *p* < 0.0001; patient 5: *Z* = −3.64, *p* < 0.001]. Matched control subjects did not show any significant deviation from the veridical mid-number for horizontal lines [*p* = 0.95, n.s., see Fig. 2A].

A regression analysis indicated that in all patients the relationship between the right bias and the length of the mental number line was significant, such that the right bias consistently increased as the length of the number line increased [patient 1: *R*^2^ = 0.044, *F*(1,70) = 3.22, *p* = 0.007; patient 2: *R*^2^ = 0.61, *F*(1,70) = 4.556, *p* < 0.03; patient 3: *R*^2^ = 0.14, *F*(1,70) = 11.15, *p* = 0.001; patient 4: *R*^2^ = 0.11, *F*(1,70) = 9.14, *p* = 0.003; patient 5: *R*^2^ = 0.21, *F*(1,39) = 11.1, *p* < 0.001]. The slope of the regression lines was significantly positive for all patients suggesting larger rightward bias as the number interval increased [patient 1: slope = 0.106, *t* = 1.794, *p* < 0.008; patient 2: slope = 0.21, *t* = 2.134, *p* < 0.04; patient 3: slope = 0.183, *t* = 3.339, *p* < 0.001; patient 4: slope = 0.181, *t* = 3.02, *p* < 0.003; patient 5: slope = 0.27, *t* = 3.33, *p* < 0.002].

Compared to control subjects, patients’ performance in bisecting horizontal number lines was significantly more biased [patient 1: *Z* = −3.94, *p* < 0.001; patient 2: *Z* = −7.37, *p* < 0.01; patient 3: *Z* = −3.291, *p* < 0.001; patient 4: *Z* = −3.45, *p* < 0.001; patient 5: *Z* = −2.36, *p* < 0.02].

#### 5.1.2 Vertical lines

All patients made errors in mentally bisecting number lines vertically-oriented (patient 1: 33%; patient 2: 40%; patient 3: 36%; patient 4: 10%; patient 5: 13%). Non-parametric tests indicated that patients 1–3 showed a significant bias towards the symbolic upper part of the line, while patients 4 and 5 showed no deviation away from the veridical mid-number [patient 1: *Z* = −1.69, *p* < 0.04; patient 2: *Z* = −7.48, *p* < 0.001; patient 3: *Z* = −2.7, *p* < .006; patient 4: *Z* = −1.74, *p* = 0.08; patient 5: *Z* = −1.73, *p* = 0.8]. Matched control subjects did not show any significant deviation from the veridical mid-number for vertical lines (*p* = 0.60, n.s., see Fig. 2B).

A regression analysis showed that in patients 1–3 the relationship between the up bias and the length of the mental number line was significant, such that up bias consistently increased as the length of the number line increased [patient 1: *R*^2^ = 0.046, *F*(1,70) = 3.38, *p* = 0.03; patient 2: *R*^2^ = 0.186, *F*(1,70) = 15.98, *p* = 0.001; patient 3: *R*^2^ = 0.89, *F*(1,70) = 6.873, *p* = 0.01]. However, this relationship was not significant for patients 4 and 5 [patient 4: *R*^2^ = 0.013, *F*(1,70) = 0.921, n.s.; patient 5: *R*^2^ = 0.163, *F*(1,31) = 5.823, *p* = 0.22, n.s.]. The slope of the regression lines was significantly positive for patients 1–3 suggesting larger up bias as the number interval increased [patient 1: slope = 0.096, *t* = 2.57, *p* < 0.011; patient 2: slope = 0.175, *t* = 3.998, *p* < 0.001; patient 3: slope = 0.16, *t* = 2.622, *p* < 0.01]. However, the slope of the regression line was not significant for patients 4 and 5 [patient 4: slope = 0.36, *t* = 0.96, *p* = 0.34, n.s.; patient 5: slope = 0.17, *t* = 2.413, *p* = 0.22].

Compared to control subjects, performance of patients 1–3 in bisecting vertical number lines was significantly more biased [patient 1: *Z* = −2.28, *p* < 0.02; patient 2: *Z* = −6.62, *p* < 0.001; patient 3: *Z* = −2.94, *p* < 0.003]. In contrast, patients 4 and 5 did not show any significant difference with controls [patient 4: *Z* = −1.38, *p* = 0.17, n.s.; patient 5: *Z* = −0.36, *p* = 0.72, n.s.].

There was no significant difference in bisecting horizontal and vertical mental number lines in three out of our five patients [patient 1: *Z* = −1.03, *p* = 0.3; patient 2: *Z* = −0.17, *p* = 0.87; patient 3: *Z* = −0.27, *p* = 0.78]. In other words, these three patients showed neglect for both horizontal and vertical lines. In contrast, a significant difference between horizontal and vertical lines was found in the other two patients, patient 4 [*Z* = −2.72, *p* = 0.006] and patient 5 [*Z* = −3.37, *p* < 0.001].

There was no significant difference in performing horizontal and vertical number lines in control subjects [*p* = 0.4, n.s.]. Horizontal and vertical lines significantly correlated [*r*(48) = 0.23, *p* = 0.05]. Moreover, the analysis of RTs showed no significant main effect of line orientation [*F*(1,11) = 3.639, *p* = 0.12, n.s.], suggesting that there was no difference in performing horizontal and vertical number lines in control subjects.

#### 5.1.3 Summary

All patients showed a bias in bisecting mental number lines symbolically oriented horizontally. This increased with the length of the line, consistent with past studies of number line bisection in neglect (Doricchi et al., 2005; Zorzi et al., 2002). Conversely, only patients 1–3 but not patients 4 and 5 showed an upward bias in bisecting number lines symbolically oriented vertically. No bias was found in bisecting number lines in control subjects.

### 5.2 Task 2: physical line bisection

#### 5.2.1 Horizontal lines

**Fig. 3:**
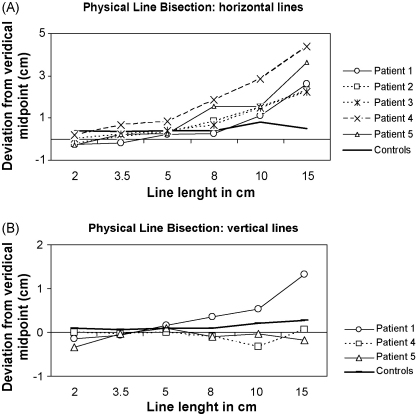
Physical line bisection task (Task 2). Patients’ and control subjects’ deviations from the veridical midpoint in horizontal (A) and vertical (B) physical lines in cm.

Non-parametric tests indicated that each patient was significantly biased overall towards the right of horizontally oriented lines [patient 1: *Z* = −2.55, *p* < 0.01; patient 2: *Z* = −4.57, *p* < 0.001; patient 3: *Z* = −6.34, *p* < 0.001; patient 4: *Z* = −4.9, *p* < 0.001; patient 5: *Z* = −2.81, *p* < 0.005]. Matched control subjects did not show any significant deviation from the veridical midpoint for horizontal physical lines (*p* = 0.4, n.s., see Fig. 3A).

A linear regression analysis indicated that for all patients the relationship between deviation and line length was significant, such that when line length increased, the rightwards bias also consistently increased [patient 1: *R*^2^ = 0.45, *F*(1,52) = 43.07, *p* < 0.001; patient 2: *R*^2^ = 0.54, *F*(1,59) = 70.19, *p* < 0.001; patient 3: *R*^2^ = 0.59, *F*(1,66) = 96.54, *p* < 0.001; patient 4: *R*^2^ = 0.83, *F*(1,30) = 143.24, *p* < 0.001; patient 5: *R*^2^ = 0.52, *F*(1,9) = 8.73, *p* < 0.02]. The slope of the regression lines was significantly positive for all patients in the horizontal line condition [patient 1: slope = 0.146, *t* = 6.56, *p* < 0.001; patient 2: slope = 0.197, *t* = 8.378, *p* < 0.001; patient 3: slope = 0.173, *t* = 9.826, *p* < 0.001; patient 4: slope = 0.328, *t* = 11.97, *p* < 0.001; patient 5: slope = 0.31, *t* = 2.96, *p* < 0.02].

A direct comparison between patients and control subjects revealed a significant difference between their performance [patient 1: *Z* = −2.28, *p* < 0.02; patient 2: *Z* = −6.62, *p* < 0.001; patient 3: *Z* = −2.94, *p* < 0.003; patient 4: *Z* = −3.45, *p* < 0.001; patient 5: *Z* = −2.13, *p* < 0.02].

#### 5.2.2 Vertical lines

Patients 2 and 3 could not perform physical line bisection task with vertically oriented lines. With these lines, only patient 1 showed a significant bias towards the upper part of the line [*Z* = −2.13, *p* < 0.03], while patients 4 and 5 showed no consistent deviation away from the veridical midpoint [patient 4: *Z* = −1.48, *p* = 0.14, n.s.; patient 5: *Z* = −0.31, *p* = 0.75, n.s.]. Matched control subjects did not show any significant deviation from the veridical midpoint for vertical physical lines (*p* = 0.2, n.s., see Fig. 3B).

A linear regression analysis indicated that for patient 1 the relationship between deviation and line length was significant, such that when line length increased, the upwards bias also consistently increased [*R*^2^ = 4, *F*(1,64) = 42.61, *p* < 0.001]. However, this was not the case for patients 4 and 5 [patient 4: *R*^2^ = 0, *F*(1,27) = 0.003, *p* = 0.96, n.s.; patient 5: *R*^2^ = 0.01, *F*(1,12) = 0.15, *p* = 0.71, n.s.]. The slope of the regressor lines indicated that in the vertical line condition this was significant only for patient 1 [slope = 0.11, *t* = 6.53, *p* < 0.001], and not for 4 and 5 [patient 4: slope = 0.0004, *t* = 0.055, *p* = 0.96, n.s.; patient 5: slope = −1.40, *t* = −0.39, *p* = 0.71, n.s.].

Performance of patient 1 in bisecting vertical physical lines was significantly more biased than controls [*Z* = 1.157, *p* < 0.03]. In contrast, patients 4 and 5 did not show any significant difference with controls [patient 4: *Z* = 0.35, *p* = 0.74, n.s.; patient 5: *Z* = 0.95, *p* = 0.39, n.s.].

Non-parametric tests showed a significant overall difference in bias between horizontal and vertical lines for patients 4 [*Z* = −4.67, *p* < 0.001] and 5 [*Z* = −2.25, *p* < 0.02] but not for patient 1 [*Z* = −0.72, *p* < 0.47, n.s]. There was no significant difference in performing horizontal and vertical physical lines in control subjects [*p* = 0.27, n.s.]. Horizontal and vertical lines significantly correlated [*r*(72) = −0.27, *p* = 0.02].

When directly compared, there was no significant difference between the two bisection tasks both in controls and in patients for horizontal (controls *t*(71) = −1.05, *p* = 0.29; all patients *p* < 0.4) and vertical lines (controls *t*(71) = −1.18, *p* = 0.24, all patients *p* < 0.2).

#### 5.2.3 Summary

All patients were biased in bisecting physical lines horizontally oriented, with a bias increasing with the length of the line. This indicates that there was a reliable relationship between bisector position and line length, consistent with past studies of line bisection in neglect (e.g. Halligan & Marshall, 1988; Harvey, Milner, & Roberts, 1995). However, only patient 1 but not patients 4 and 5 showed an upward bias in bisecting physical lines vertically oriented. No bias was found in performing physical lines in control subjects, and no difference between number lines and physical lines.

### 5.3 Task 3: landmark

**Fig. 4:**
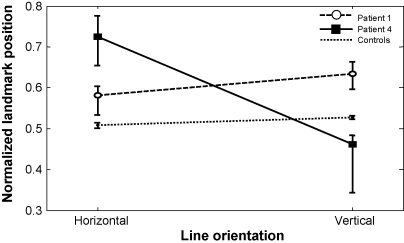
Estimated PSE values and their 95% confidence limits for patients 1 and 4 and control subjects. Separate data-points are shown for horizontal and vertical line conditions, pooled across quadrants and blocks.

In Task 3, data were pooled across blocks and quadrants, after having first established that there were no consistent differences between the four positions around the fixation point at which a line could appear. Fig. 4 graphs the position of the subjective midpoint (i.e. PSE values), for the patients 1 and 4 separately, with 95% confidence intervals (computed for each data point using the method described in Appendix B) for horizontal and vertical line orientations. Fig. 4 also shows the means for six matched controls with error bars indicating the 95% confidence interval, based on their standard error. Values higher than 0.5 indicate that the subjective midpoint was biased towards the right or upper ends of the horizontal or vertical lines, respectively.

For patient 1 (see open symbols in Fig. 4), overlapping error-bars for the horizontal and vertical conditions indicate no significant difference between line orientation conditions. Conversely, error bars for patient 4 (filled symbols) are clearly separate for horizontal and vertical conditions, indicating a significant difference between line orientation conditions. The confidence limits attached to each PSE estimate also allowed an assessment of whether each patient's subjective midpoint was significantly biased away from the veridical midpoint of the lines (0.5 in the graphs) and also from the mean PSE of controls. These are displayed on the graphs as dot symbols with 95% confidence limits based on the standard error of the mean PSE across control subjects. Significant rightwards and upwards biases (*p* < 0.05) from veridical midpoint and control PSE were observed only for patient 1, while for patient 4 only a rightwards bias with horizontal lines was clearly significant.

There was no significant difference in RTs between horizontal and vertical lines in control subjects’ performance [*t*(6) = 1.46, *p* = 0.19, n.s.].

## 6 Discussion

This study aimed at exploring the mechanisms operating in number and physical line bisection in five patients with unilateral neglect. The performance of three patients (patients 1–3) remained unchanged when numbers where oriented horizontally, such as houses along a street, or vertically, such as floors in a house. All three patients showed a similar bias consisting of a shift towards the right in the case of horizontal number lines and upward for vertical ones. For instance, when asked to state the middle number between ‘1’ and ‘5’ these patients typically said ‘4’. We could assess bisection of physical lines in only one of these three patients (patient 1). Interestingly, she presented the same bias in bisecting physical lines as when bisecting mental number lines. When asked to bisect a horizontal physical line, she showed a rightward bias; when asked to bisect a vertical physical line she showed an upward bias.

In striking contrast, the remaining two neglect patients (patients 4 and 5) showed a dissociation between horizontal and vertical bias. Specifically, they presented a similar rightward bias in bisecting both physical and mental number lines that were horizontally oriented. However, they showed no such bias in bisecting either physical or mental number lines that were vertically oriented. The results of our neuropsychological assessment do not allow us to draw any conclusion regarding whether some specific focal cognitive deficit was present in patients with or without perceptual and representational vertical neglect.

We analyzed the magnitude of the patients’ bias. Previous research has indicated that in patients with neglect the magnitude of the bias increases with the length of the horizontally presented physical and mental number lines (e.g. Bisiach & Vallar, 1988; Halligan & Marshall, 1988; Harvey et al., 1995; Priftis et al., 2006; Zorzi et al., 2001). So for example, patients indicated that ‘4’ is the middle number between ‘1’ and ‘5’ and that ‘8’ is the middle number between ‘1’ and ‘9’. No data have been reported until now for vertically presented physical and number lines. In all of our patients we found that whenever a bias occurred (whether horizontal or vertical) there was the same incremental pattern. Thus these findings replicate and extend previously reported magnitude effects in biased performance in neglect patients for horizontal to vertical bisection of physical and mental number lines.

We compared the patients and control subjects performance in the two bisection tasks and in the horizontal and vertical dimensions. No difference was found between the two bisection tasks (numbers and physical lines) nor between the two dimensions in control subjects’ performance. Moreover, no difference in response times was found between horizontal and vertical lines in number bisection and in the landmark task in control subjects. Therefore, it is unlikely that the horizontal and vertical dimensions differ in principle in terms of difficulty level. Equally unlikely is the possibility that any dissociation between horizontal and vertical dimensions reflects different levels of familiarity of their mental representations. Indeed, these representations do not seem required in bisecting physical lines. Nevertheless, two of our patients showed a dissociation between horizontal and vertical physical lines.

We will discuss our patients’ impairment in bisecting physical and number lines first in the context of the classical neglect literature and secondly within more recent theoretical accounts proposed for neglect of mental number line. Only a few studies investigated the performance of neglect patients in bisecting horizontal and vertical physical lines. The majority of these studies documented an association of deficits, namely patients were equally impaired when processing horizontal and vertical lines (e.g. Ergun-Marterer et al., 2001; Halligan & Marshall, 1989, 1991; Ladavas et al., 1994; Mark & Heilman, 1997). Similarly to our patients, the majority of patients with vertical neglect showed an upward bias (e.g. Bender and Teuber, 1948; Rapcsak et al., 1988; Shelton et al., 1990). One of our patients (patient 1) seemed consistent with this pattern of performance as she showed similar horizontal and vertical neglect for both physical and number lines. Only a few studies showed a dissociation between horizontal and vertical line bisection, reporting a selective impairment for either horizontal or vertical lines (e.g. Bender & Teuber, 1948; Ergun-Marterer et al., 2001; Milner & Harvey, 1995; Pitzalis et al., 1997; Rapcsak et al., 1988; Shelton et al., 1990). Two of our patients showed a selective impairment for horizontal both physical and number lines. This suggests that in both patients the whole vertical dimension was intact whereas the left horizontal one was impaired. The pattern of performance documented in our patients therefore confirms that horizontal and vertical neglect can associate or dissociate among different patients.

Two main proposals have been put forward to account for neglect in mental number line (Doricchi et al., 2005; Zorzi et al., 2002). Interestingly, both proposals suggest that there are (at least partially) common mechanisms between physical and numerical representations. In particular, it has been suggested that the mental number line is spatially organized in the horizontal dimension with small numbers on the left side and large numbers on the right side (Doricchi et al., 2005; Zorzi et al., 2002; see also Dehaene et al., 1993; Hubbard et al., 2005). Both accounts also propose that the mental number line can be impaired following brain damage. The similar performance in physical and mental number line bisection that we observed in our neglect patients further supports the idea that physical and numerical representations have some mechanisms in common.

However, the dissociation we documented between horizontal and vertical mental number line bisection creates interesting problems for both these proposals. Doricchi et al. (2005) suggested that when bisecting mental number lines one needs to covertly navigate along the line. This navigation requires working memory mechanisms which are different from those required to navigate along physical lines. If working memory mechanisms are required to navigate along the mental number line then one might expect similar patterns of performance between horizontal and vertical number line bisection. However, two of our neglect patients showed selective impairment only in horizontal number line bisection. Moreover, none of our patients showed any sign of working memory impairment despite an impaired performance in number line bisection. Therefore, working memory mechanisms may be necessary but not sufficient for navigation along the number line.

Zorzi et al. (2002) suggested that mental number bisection operates on an internal representation analogous (or isomorphic) to a horizontal physical line. This hypothesis predicts a similar bias in physical and mental number lines. All our patients were in agreement with this prediction when bisecting horizontal lines as they showed a similar bias for physical and number lines. However, the data of two of our patients (4 and 5) do not confirm this prediction. In both patients neglect was present for horizontal but not for vertical lines. This implies that the internal representation on which the number line operates may be analogous to physical lines of a variety of orientations, not just horizontal as initially assumed.

We would like to suggest that our data lend empirical support to the notion that there may be also a vertical number line. This vertical number line appears to be organized with small numbers at the bottom and large numbers at the top. The three patients (1–3) with vertical neglect consistently neglected the symbolic lower part of the vertical number line. This supports the idea that the vertical number line has a bottom-to-top orientation. Additional evidence of bottom-to-top orientation of the vertical number line comes both from the introspection of people with number-forms, also referred to as a form of synaesthesia (Galton, 1880; Sagiv, Simner, & Collins, 2006; Seron, Pesenti, & Noël, 1992) and from behavioural studies (e.g. Gevers et al., 2006; Ito & Hatta, 2004; Schwarz & Keus, 2004). For instance, Ito and Hatta (2004) showed the existence of the SNARC effect in the vertical dimension, where subjects are faster to respond to small numbers in the lower part of the space and large numbers in the upper part of the space (Ito & Hatta, 2004). Altogether, this evidence suggests that vertical number lines are bottom-to-top organized and that bias in bisecting these lines affects the lower part of the line. Thus, we suggest that number line bisection operates on internal horizontal and vertical representations analogous to horizontal and vertical physical lines.

Our study not only provides evidence of the existence of a vertical mental number line in neglect patients but also allows us to speculate about the relationship between horizontal and vertical number lines. We have shown that patients can have a selective impairment in the horizontal number line. This suggests that the vertical number line can successfully operate independently from the horizontal line. Thus, at least partially independent cognitive mechanisms appear to be involved in processing horizontal and vertical number lines. Further research is needed to elucidate whether the horizontal number line can also operate independently from the vertical one.

What are the anatomical bases of horizontal and vertical number lines? So far, there have been no proposals of the anatomical bases of vertical number line processing. On the other hand, some authors suggested that operating along horizontal number line depends primarily on mechanisms located in or around the parietal areas (e.g. Doricchi et al., 2005; Hubbard et al., 2005). In addition, it has also been suggested that navigating along the mental number line relies on spatial working memory mechanisms located in the frontal areas (Doricchi et al., 2005).

Four out of five of our neglect patients showed large lesions involving mainly, although not exclusively, the right parietal lobe that in two cases extended anteriorly (patients 3 and 4). In one case (patient 1) there was no parietal lesion but the right temporal lobe was involved instead. Our anatomical data do not allow us to draw any firm conclusion regarding the involvement of different lesions sites in patients with and without perceptual and representational vertical neglect. We note that the data published so far also do not allow us to draw firm conclusions regarding the neuroanatomical correlates. Perceptual neglect is thought to be mainly associated with right parietal lesions (e.g. Heilman, Watson, & Valenstein, 2003; Driver & Vuilleumier, 2001). However, it also occurs following lesions to the inferior frontal and superior temporal cortex and subcortical areas (e.g. Vallar, 2001). Representational neglect has been suggested to rely mainly on temporal regions (e.g. Bisiach & Luzzatti, 1978). However, patients with selective representational neglect following lesions to other areas have also been reported (e.g. Guaraglia, Padovani, Pantano, & Pizzamiglio, 1993; Ortigue et al., 2001). It has also been suggested that processing horizontal and vertical physical lines share some anatomical networks, mainly located in the right inferior parietal cortex (Fink, Marshall, Weiss, & Zilles, 2001). Nevertheless, clinical and imaging studies suggested that bisection of horizontal physical lines is also associated with the striate and extrastriate visual cortex and with the right superior parietal lobe (Doricchi et al., 2005; Fink et al., 2000).

In conclusion, the present study provides evidence that processing physical and mental number lines can dissociate depending on whether they are oriented horizontally or vertically. Our data suggest the existence of relatively independent horizontal and vertical lines. Right parietal and temporal regions appear to be involved in processing horizontal and vertical lines although there is clearly need to clarify their role in number and space processing.

## Figures and Tables

**Fig. A.1:**
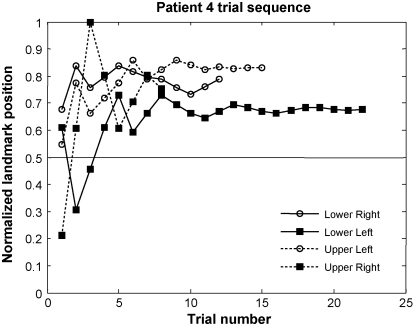
Typical trial sequence for one block in Experiment 2, illustrating the function of the adaptive algorithm (patient 4 in horizontal condition) for each of the quadrants of stimulus presentation. The landmark switches between leftwards and rightwards offsets (negative and positive values on the *y*-axis, respectively) each time the subject's ‘left’ vs. ‘right’ responses change, making progressively smaller increments with each successive switch, until finally converging on the subjective midpoint at which either of the ‘left’ or ‘right’ responses were equally likely. In this example the convergence points are shifted towards the right of the horizontal line.

**Fig. A.2:**
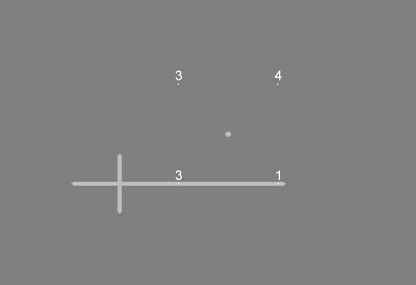
An example stimulus used in the landmark task. The dot indicates where participants should fixate their gaze. The four numbers indicate the possible positions in one of four quadrants of the screen (upper left, upper right, lower left and lower right), fixed at 1.15° eccentricity from central fixation. The veridical midpoint of each long line was always centred on one of these four positions (measured not in scale).

**Fig. A.3:**
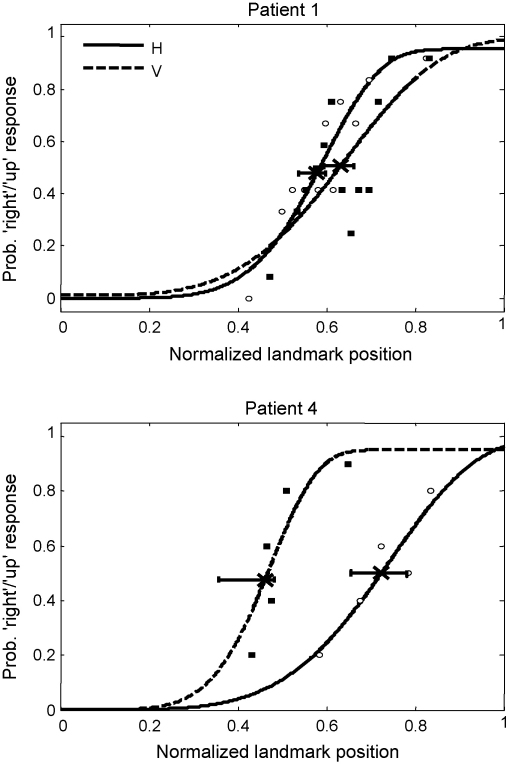
Psychometric functions and least-square fits for the two patients tested with Experiment 3. See Appendix B for detailed explanation.

**Table 1:** Summary of the patients’ cognitive scores (number correct; percentiles are reported in brackets)

Tasks performed	Patient 1	Patient 2	Patient 3	Patient 4	Patient 5
General intellectual abilities					
WAIS-R verbal I.Q.	79	89	74	96	67
WAIS-R performance I.Q.	62	n.t.	n.t.	n.t.	n.t.
Coloured progressive matrices	n.t.	20/36 (80–90)	14/36 (70–80)	0/36^a^	n.t.

Memory					
Recognition memory test					
Faces	11/25 (<5th %ile)	n.t.	8/25 (<5%ile)	n.t.	6/25 (<5%ile)
Words	23/25 (>25th %ile)	36/50 (10–25th %ile)	25/25 (75th %ile)	15/25 (<5th %ile)	20/25 (75th %ile)

Digit span	6	7	6	5	6

Picture naming	13/30 (O)	21/30 (GNT, 50–75%ile)	21/30 (O)	13/30 (O)	21/30 (O)

Executive functions					
Phonological fluency (‘S’)^b^	1 (<5% cut-off)	6 (<5% cut-off)	7 (<5% cut-off)	n.t.	6 (<5% cut-off)

Visuo-perceptual and visual–spatial functions					
Incomplete letters	16/20 (>5% cut-off)	13/20 (<5% cut-off)	15/20 (<5% cut-off)	9/20 (<5% cut-off)	15/20 (<5% cut-off)
Position discrimination	n.t.	10/20 (<5% cut-off)	10/20 (<5% cut-off)	n.t.	10/20 (<5% cut-off)

Neglect					
Star cancellation test	L = 0/26; R = 8/26	L = 6/26; R = 24/26	L = 9/26; R = 24/26	L = 5/26; R = 26/26	L = 4/26; R = 22/26
Object drawing	0/3	1/3	1/3	1/3	0/3
Line bisection (deviation to *R^*)	0.63 mm	0.81 mm	0.84 mm	0.80 mm	0.48 mm

n.t.: not tested. O: Oldfield naming test; GNT: graded naming test; L: left-hand side of the paper; R: right-hand side of the paper. *R*^: right. In brackets standardized score.

a Unable to engage in the task.

b Number of items produced in 1 min.

## Appendix A Modified binary search algorithm

The goal of this algorithm was to quickly converge on the subjective midpoint of the line, defined as the position at which subjects’ responses (‘left’/‘right’ or ‘up’/‘down’ for horizontal and vertical lines, respectively) are at equal probability. For example, say that on the first trial of a block, the stimulus was positioned in the upper left quadrant, and the subject indicates that the landmark appeared ‘right’ of the midpoint. In the next trial for the upper left quadrant, the Landmark would be physically offset by a set increment to the left relative to its previous position (see Tyrrell and Owens, 1988 for a fuller explanation of how increment size is controlled). Note that this second trial for the upper left quadrant could be preceded by other trials for the other three quadrants, as four independent algorithms were interleaved in pseudo-random order for each quadrant. If in this second trial the subject's response was now ‘right’, i.e. a response ‘reversal’, the landmark would next shift left, but by a smaller amount than before. Conversely, if the subject persisted in responding ‘left’, the landmark would begin to shift rightwards by increasing amounts. The algorithm would terminate when the size of the increment became smaller than a criterion distance, set in this experiment to 1% of the total line length, or when the total number of response reversals exceeded a limit, set here to 10. To illustrate the operation of this algorithm, Fig. A.1 graphs a typical protocol for one block (for subject AS in the horizontal condition). Separate trial sequences are shown for each of the four quadrants, plotting landmark position on the *y*-axis against trial number, Fig. A.2 shows an example stimulus used in the task.

## Appendix B Bootstrapping method

In order to obtain an estimate of the variance of subjects’ PSE's in Experiment 2, we first took the raw trial-by-trial data (compiled across blocks and quadrants) and calculated the probability of a ‘right’/‘up’ versus ‘left’/‘down’ response as a function of the different physical landmark positions. Plotting these probabilities against landmark position revealed an s-shaped ‘psychometric function’ of the kind typically observed in psychophysics (see the dots in Fig. A.2). In the present case this took the following form for all participants: as the landmark was offset towards the right or the upper end of a line, the probability of ‘right’ or up’ responses tended to increase (see circle and square datapoints for horizontal and vertical line orientations, respectively). The degree to which the subjective midline was biased in the patients could be assessed by examining the characteristics of this psychometric function. With horizontal lines, for example, if the left space is neglected, the subjective midpoint is typically shifted towards the right; consequently patients should be more likely to report that the landmark appeared ‘left’ of their perceived midpoint, thus shifting the psychometric curve towards the right. Using standard psychophysics methods, we used a least-squares procedure to fit the individual participants’ data with a Weibull function (Wichmann & Hill, 2001a, 2000b; see continuous and dotted lines in Fig. A.2, for horizontal and vertical conditions, respectively). The parameters describing the Weibull function were then used to estimate the PSE mathematically. In Fig. A.3 these estimated PSE values typically correspond to the point along the curves at which they are straightest.

To obtain an error estimate for the PSE, we used a ‘bootstrapping’ procedure (PSIGNIFIT for Matlab, http://www.bootstrap-software.org/psignifit/publications.php, Wichmann & Hill, 2001a, 2001b). This simulates 1000 virtual datasets defined by the same statistical properties of the actual dataset, and fits each of them to obtain a range PSE estimates. The mean and standard error across these different estimates may then be calculated. These error estimates (95% confidence limits) are represented graphically by horizontal error-bars in Fig. A.2 around the estimated PSE, and are reproduced in Fig. 4.
